# Biphasic dendritic growth of dorsolateral prefrontal cortex associative neurons and early cognitive development

**DOI:** 10.3325/cmj.2018.59.189

**Published:** 2018-10

**Authors:** Dora Sedmak, Branka Hrvoj-Mihić, Domagoj Džaja, Nikola Habek, Harry B. M. Uylings, Zdravko Petanjek

**Affiliations:** 1 Department of Anatomy and Clinical Anatomy, University of Zagreb School of Medicine, Zagreb, Croatia; 2 Croatian Institute for Brain Research, University of Zagreb School of Medicine, Zagreb, Croatia; 3 Department of Anthropology, University of California San Diego, La Jolla, CA, USA; ^4^Department of Physiology and Immunology, University of Zagreb School of Medicine, Zagreb, Croatia; 5 Department of Anatomy and Neuroscience, Graduate School of Neurosciences Amsterdam, Amsterdam University Medical Center, Amsterdam, the Netherlands; * DS and BHM contributed equally

## Abstract

**Aim:**

To analyze postnatal development and life-span changes of apical dendrite side branches (oblique dendrites) from associative layer IIIC magnopyramidal neurons in the human dorsolateral prefrontal cortex and to compare the findings with the previously established pattern of basal dendrite development.

**Methods:**

We analyzed dendritic morphology from 352 rapid-Golgi impregnated neurons (10-18 neurons per subject) in Brodmann area 9 from the post-mortem tissue of 25 subjects ranging in age from 1 week to 91 years. Data were collected in the period between 1994 and 1996, and the analysis was performed between September 2017 and February 2018. Quantitative dendritic parameters were statistically analyzed using one-way analysis of variance and two-tailed *t* tests.

**Results:**

Oblique dendrites grew rapidly during the first postnatal months, and the increase in the dendrite length was accompanied by the outgrowth of new dendritic segments. After a more than one-year-long “dormant” period of only fine dendritic rearrangements (2.5-16 months), oblique dendrites displayed a second period of marked growth, continuing through the third postnatal year. Basal and oblique dendrites displayed roughly the same growth pattern, but had considerably different topological organization in adulthood.

**Conclusion:**

Our analysis confirmed that a biphasic pattern of postnatal dendritic development, together with a second growth spurt at the age of 2-3 years, represents a unique feature of the associative layer IIIC magnopyramidal neurons in the human dorsolateral prefrontal cortex. We propose that these structural changes relate to rapid cognitive development during early childhood.

Neuronal circuitries of the cerebral cortex form anatomical and functional foundations for complex cognitive, motor, and sensory abilities (1,2). Even minor changes in the organization of neural circuitry during development influence the function of cortical networks in the adulthood, often leading to major disorders (3,4). An essential process in neuronal circuitry differentiation and maturation is the development of dendrites, which represent the major receptive field of neurons (5). Dendritic development is regulated by various intrinsic, environmental, and epigenetic factors (6-10), all of which influence the formation of neuronal connections, and consequently the functioning of the neuronal network.

For most subpopulations of pyramidal neurons in the human cerebral cortex, the main period of dendritic growth takes place within the first postnatal year (11-15). An important exception are the large layer III pyramidal neuronal (L3N) population in the dorsolateral prefrontal cortex (DLPFC). Basal dendrites on L3N in the DLPFC display a unique developmental pattern characterized by two growth spurts separated by a year-long period of stasis (1). The DLPFC is the region affected by most intensive structural and functional changes related to protracted brain maturation throughout childhood and adolescence (16,17). It is also involved in many complex mental functions, as shown by *in-vivo* imaging studies (18,19). These structural changes are related to the rapid maturation of higher-order cognitive abilities around the age of two, such as the development of the initial elements of the theory of mind (20). The second growth spurt of basal dendrites occurring at that age may be related to selective reorganization and maturation of cortical microcircuitries responsible for more efficient processing throughout the cortical network (21). The main efferent inter- (22) and intra-areal (23) projections of L3N characterize them as associative neurons. Experimental studies on non-human primates have confirmed that they are the key elements involved into working memory and other higher cognitive functions processed in the DLPFC (24). These findings strongly suggest that the L3N maturation in the DLPFC is a major event in extensive cognitive development during early childhood.

Since apical oblique and basal dendrites differ in the origin of their afferents and respond differently to alterations of afferent inputs (25-28), it is important to analyze the development of the two types of dendritic trees separately. In addition, existing research suggests that oblique and basal dendrites are affected differently in neurological and psychiatric conditions (29-32). The aim of this study was to quantitatively and qualitatively analyze changes in the length and complexity of oblique dendrites from associative L3N in the DLPFC (Brodmann area 9) and compare the findings with the developmental pattern previously described for basal dendrites (1). We hypothesized that the differences in maturation between the thalamic, cortical, and intracortical systems would be reflected in differences in maturation between apical oblique and basal dendrites.

## Materials and methods

Dendritic morphology was analyzed on cortical samples of 25 subjects with no history of psychiatric and neurological disorders, encompassing 352 neurons in total, that is, 10-18 L3N per subject. Subjects' age ranged from 1 week to 91 years (Table 1). The time interval between death and tissue fixation (ie, postmortem interval; PMI) was shorter than 8 h for early postnatal subjects, shorter than 13 h for infants, shorter than 16 h for children, and shorter than 20 h for adults. All subjects died without preagonal state, so that the PMI represents the interval between the actual neuronal death and fixation. No staining artifacts due to PMI, described for rapid Golgi staining (33), were detected in the analyzed subjects. Quantitative analysis did not reveal any effects of PMI on terminal segment length, which is the part of dendrites most prone to be affected by long PMI. This study used the same archival sample (34,35) previously used in the analysis of basal dendritic maturation (1). The tissue was obtained with the approval of the Ethics Committee of Zagreb University School of Medicine, and is currently regulated by Ethics Committee approval number 380-59-10106-14-55/152 from July 1, 2014.

**Table 1:** Characteristics of tissue sample

Age	Case number	Sex	Post-mortem delay (hours)	Cause of death	Section thickness (μm)	Number of neurons quantitatively studied	Percentage of incomplete segments	
							basal dendrites	oblique dendrites
1 week	cd96	F	3	bmn*	140	16	4	13
1 month	cd147	M	4	sudden infant death syndrome	160	16	5	12
2.5 months	cd105	M	6	pneumonia	140	14	8	19
7 months	cd123	M	5	sudden infant death syndrome	165	15	22	46
12 months	cd107	M	13	sudden infant death syndrome	170	14	16	39
15 months	cd143	F	5	bmn*	170	16	8	38
16 months	cd159	F	12	mucoviscidosis	165	10	12	22
2 year	cd238	F	15	neuroblastoma	200	12	18	42
2.5 years	cd175	M	16	car accident	165	16	11	38
5.5 years	cd125	F	15	strangulation	180	10	23	72
6 years	cd156	M	15	car accident	165	18	16	22
9 years	cd157	M	16	car accident	160	14	11	55
10 years	cd150	F	16	CO poisoning	175	15	14	40
16 years	co185	F	8	car accident	185	14	14	27
17 years	co170	M	20	bmn*	175	15	14	40
19 years	co167	M	20	strangulation‡	165	15	13	54
22 years	co198	M	20	strangulation‡	210	11	19	42
28 years	co192	M	6	car accident	180	12	16	37
30 years	co180	M	12	car accident	180	15	14	52
52 years	co215	F	6	car accident	200	10	12	25
59 years	91.172†	M	6	thoracic embolism	120	16	19	40
62 years	co246	F	11	lung cancer	180	15	12	30
82 years	92.01†	M	6	urogenital cancer	135	16	22	44
87 years	co171	M	8	bmn*	140	15	11	30
91 years	91.77†	M	4	cerebrovascular insult	120	12	25	48

*bmn – brain morphology normal, no psychiatric and neurological diseases present in the anamnesis, the cause of death not described in the protocol.

†specimens from the Netherlands Brain Bank, Amsterdam.

‡subjects who committed suicide without evidence of psychiatric and neurologic history.

**Figure 1:**
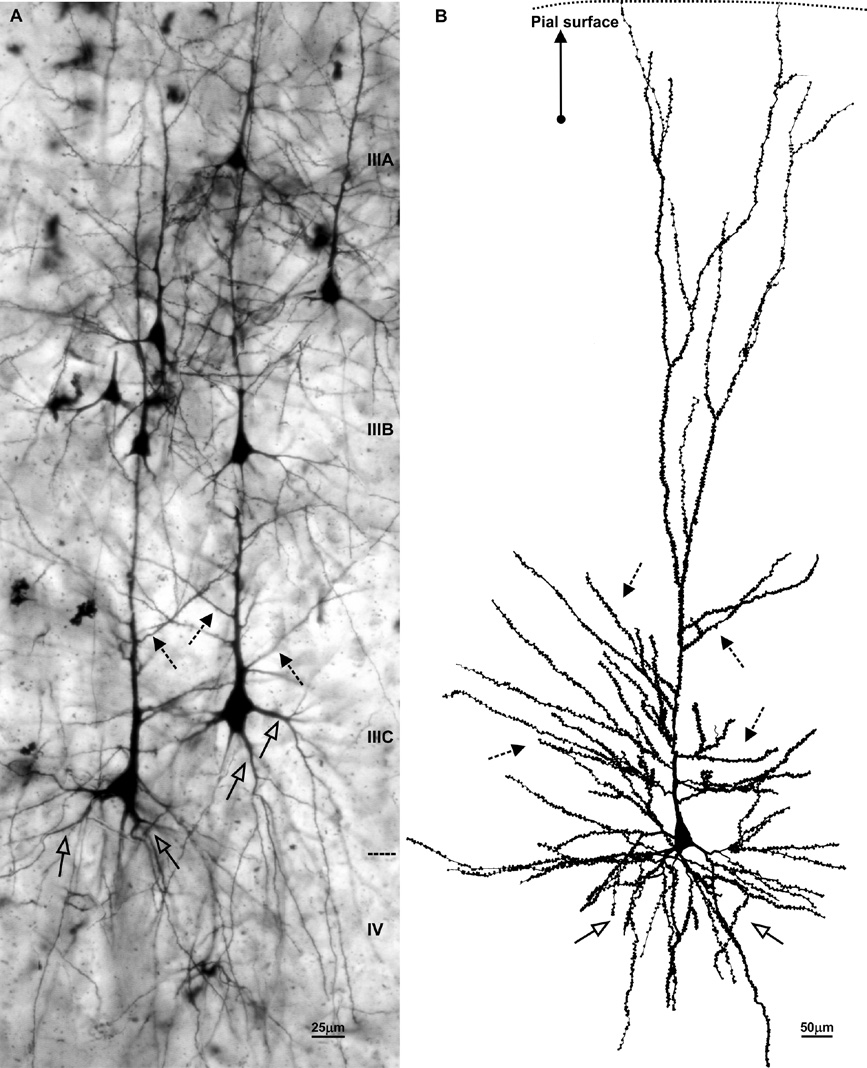
Magnopyramidal layer IIIC neurons were easily distinguished from neighboring neurons by their large cell body and prominent dendrites. Oblique dendrites were defined as branches originating from the main apical shaft, as illustrated in the microphotograph (**A**) and drawing (**B**) of a rapid Golgi impregnation from the dorsolateral prefrontal cortex (Brodmann area 9) of a 49-year-old (**A**) and 2.5-year-old subject (**B**). Arrows with open head indicate basal dendrites; dashed arrows indicate oblique dendrites (scale bar: 25 μm). At 2.5 years, dendritic tree size had reached adult values and the spine density was at the maximum.

The tissue was derived from the right hemisphere at the position of the superior and middle frontal gyrus, corresponding to the cortical region defined as frontal granular and magnopyramidal Brodmann area 9 (BA 9) (36). The samples were processed using a standard chrome-osmium rapid Golgi method (1). Cortical blocks were directly immersed into rapid Golgi solution (0.3% osmium tetroxide and 3% potassium dichromate) for 7 days, followed by immersion into 1% silver nitrate for 2 days. The tissue was then dehydrated and embedded in 8% celloidin. The blocks were sectioned on a microtome into coronal sections, mostly 160-200 μm thick. This section thickness was a compromise between cutting the dendritic systems and getting the best visibility of dendrites inside one section (37). Quantitative analysis did not reveal any effects of section thickness on the analyzed parameters. The analysis was conducted only on magnopyramidal layer IIIC neurons (Figure 1) (38). The measurements included three-dimensional reconstruction of apical dendrite main shaft and its side branches (oblique dendrites) with a semi-automatic dendrite measuring system using a 63 × oil immersion objective with a long working distance (1).

Morphometric analysis was conducted using the following variables (39): 1) total number of oblique dendrites per neuron; 2) total length of the apical dendrite main shaft including terminal bifurcations following the course of the main shaft–but without side branches, ie oblique dendrites–per neuron; 3) total length of oblique dendrites per neuron (summed length of all traced oblique dendritic segments including the length of the individual incomplete segments, ie, dendritic segments that could not be followed to their original endings); 4) total number of oblique dendritic segments per neuron (ie, number of dendritic branches); 5) mean length of oblique dendrite individual terminal and intermediate segments; 6) radial distance of individual terminal and intermediate oblique dendritic segments relative to the origin of oblique dendrite on the apical main shaft. Oblique dendrites were defined as side branches of the apical dendrite, that is, the dendrites originating under about a right angle (at least 70°) from the main apical dendritic shaft. Terminal segments were defined as segments between the terminal tip of dendrites and the last bifurcation point before the terminal tip. Intermediate segments were defined as segments between two branching points or between the dendritic origin and the next bifurcation point. The topological position of segments was indicated following centrifugal ordering (39). Data showing mean length of oblique dendritic terminal segments and radial distance of terminal segments did not include values for incomplete dendritic segments. The measurements were not corrected for tissue shrinkage.

The neuronal reconstruction was performed by two researchers (ZP performed all reconstructions and HBMU repeated the reconstructions of randomly selected neurons to verify the consistency of obtained data) from February 1, 1994 to July 1, 1998 at the Netherlands Institute for Brain Research, Amsterdam, The Netherlands. The slides were coded, so that the investigators were not aware of the subjects’ age, sex, or medical history. The analysis of reconstructed neurons was performed by two researchers (DS and BHM) from September 1, 2017 to February 1, 2018 at the Department of Neuroscience, Croatian Institute for Brain Research, School of Medicine, University of Zagreb, Zagreb, Croatia and Department of Anthropology, University of California San Diego, La Jolla, CA, United States.

### Statistical analysis

Statistical analysis was performed as reported previously (1). One-way analysis of variance (ANOVA) with parametric and nonparametric analyses was used to assess the association of age as the main effect and dendritic variables, with every subject representing a separate stage (40). The nonparametric analysis used rank transformation procedure according to Conover and Iman (41). The *a posteriori* (*post-hoc)* Student-Newman–Keuls (SNK) test for multiple comparisons was applied to determine statistical differences between subjects. Parametric and nonparametric procedures showed comparable results. Subjects were divided into groups based on their age, and morphometric values obtained for oblique dendrites were compared between subjects belonging to two consecutive age groups. Values for basal dendrites obtained in our previous study (1) were then compared with the oblique dendrite data between equivalent age groups. These comparisons were made using two-tailed *t* test (42), and mean values per subject were used as raw data. Regression analysis was used to test the existence of a pattern of increase or decrease in a series of cases. The level of significance was set at *P* = 0.05. Statistical analysis was performed using SPSS software, version 8 (licensed to the Netherlands Institute for Brain Research) (12) and STATISTICA software, version 10, (Statsoft, Tulsa, OK, USA, licensed to the University of Zagreb School of Medicine).

## Results

### Qualitative observations

From the age of 2.5 years, L3N were easily identified by their prominent soma and elaborate basal dendritic tree. They were located in the deepest part of the layer III (layer IIIC), that is, the zone 200 μm above the non-impregnated layer IV (Figure 1A). Most basal dendritic segments of L3N expanded into the layer IV, running perpendicular to the pial surface. The cell bodies of L3N generally displayed a regular shape of an isosceles triangle, with the short side on the base (parallel to the border of layer IV) and the tip on the opposite side, from where a thick apical dendrite extends toward the pial surface. The apical dendrites ran at least up to the middle of layer III without bifurcating. The main dendritic shaft usually branched 1 to 2 times before making extensive terminal ramifications of thinner dendrites through layer II and layer I. Neurons with an apical dendrite that could be followed for a longer distance typically gave rise to 6-10 side oblique dendrites (Figure 1B).

In adult subjects, all dendrites were covered with dendritic spines. Dendritic spines were not observed on the cell body, the most proximal part of the apical dendrite, or on basal dendrites close to the soma (Figure 1A). On other neuron compartments, spine density gradually increased and was highest in the middle of the apical dendrite main shaft. From there toward the tip of the apical dendrite (ie, the distal terminal tuft), spine density slowly but continuously decreased. Similarly, oblique dendrites emerging from distal parts of the apical dendrite displayed higher density of dendritic spines compared to those emerging from more proximal parts (Figure 1B). However, we did not qualitatively observe any consistent gradient in dendritic spine density on individual – either basal or oblique – dendrites.

**Figure 2:**
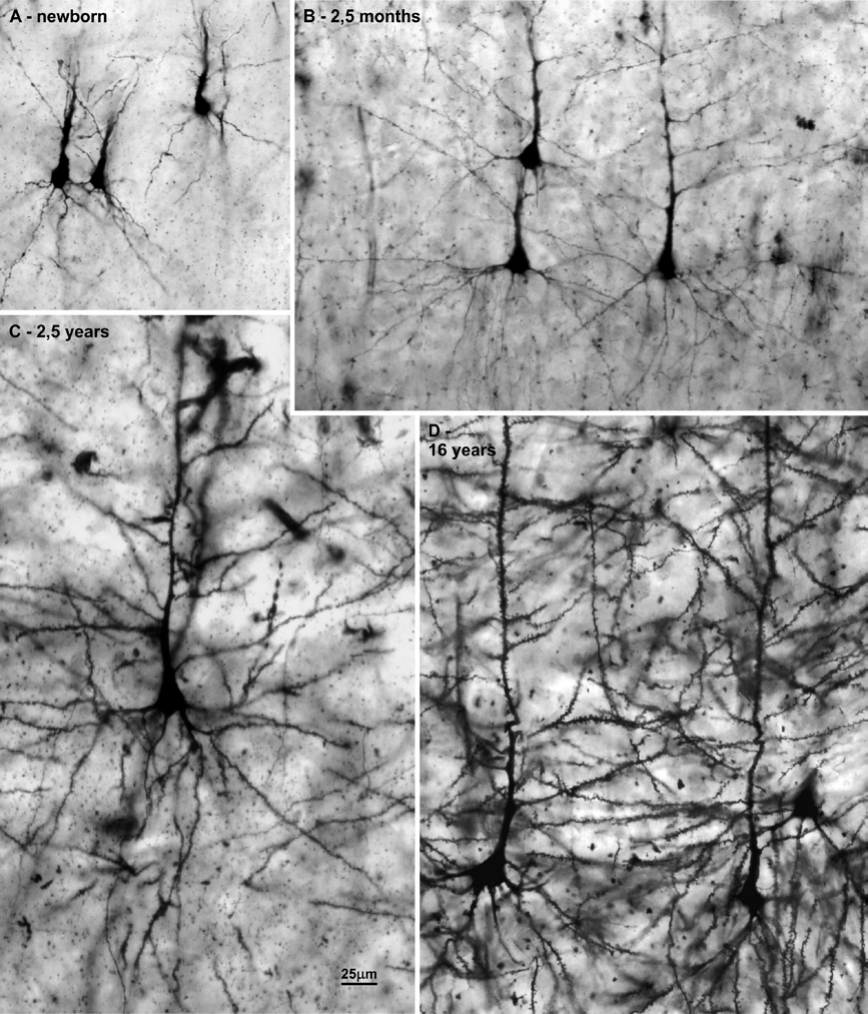
The first 2.5 postnatal months represent a period of rapid increase in complexity (ie, branching) of oblique dendrites in the dorsolateral prefrontal cortex (Brodmann area 9). This conclusion is based on the comparison of oblique dendrites between a newborn (**A**) and a 2.5-month-old infant (**B**). The dendritic tree has reached adult morphology by 2.5 years of age (**C**) and it does not differ qualitatively from the dendritic tree seen in adults (**D**); (scale bar = 25 μm.)

**Figure 3:**
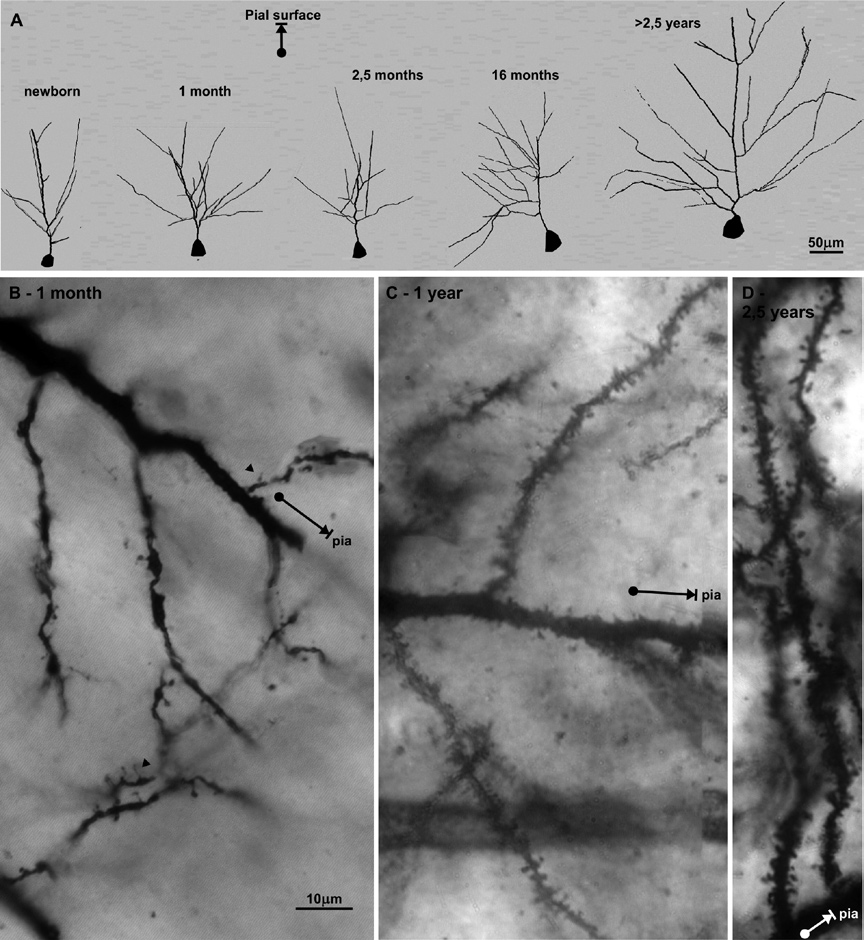
The described growth pattern is illustrated in the three-dimensional reconstruction of the apical dendrite and its side branches (oblique dendrites) of rapid Golgi impregnated layer IIIC pyramidal cells in the dorsolateral prefrontal cortex (Brodmann area 9) projected onto the coronal plane. Arrow indicates the direction of the pial surface (scale bar = 50 μm) (**A**). Note the low density of dendritic spines in the 1-month-old subject (**B**) and the increase in dendritic spine density during first year (scale bar = 10 μm) (**C**) up to 2.5 years (**D**), when most of the dendritic surface becomes covered with spines. This increase in the dendritic spine density occurs in parallel with the dendritic tree thickening.

The majority of the outgrowth of oblique dendrites occurred during the first postnatal month (Figure 2). Interestingly, thereafter dendritic elongation did not increase gradually, but instead took place in the period between 16 months and 2.5 years (Figure 3A). During the perinatal period (ie, from birth until one month of age) dendritic spines on oblique dendrites were sparse, and those that were present displayed immature, hair-like morphology (Figure 3B). In the 2.5-month-old specimen, most spines had acquired their mature mushroom form. Spine density increased up to the age of 2.5 years (Figure 3C,D), when they approached their maximum levels, and covered most of the dendrite surface. The spine density remained high throughout the rest of childhood and adolescence until the third decade, during which it dropped to its final level.

### Quantitative analysis

**Figure 4:**
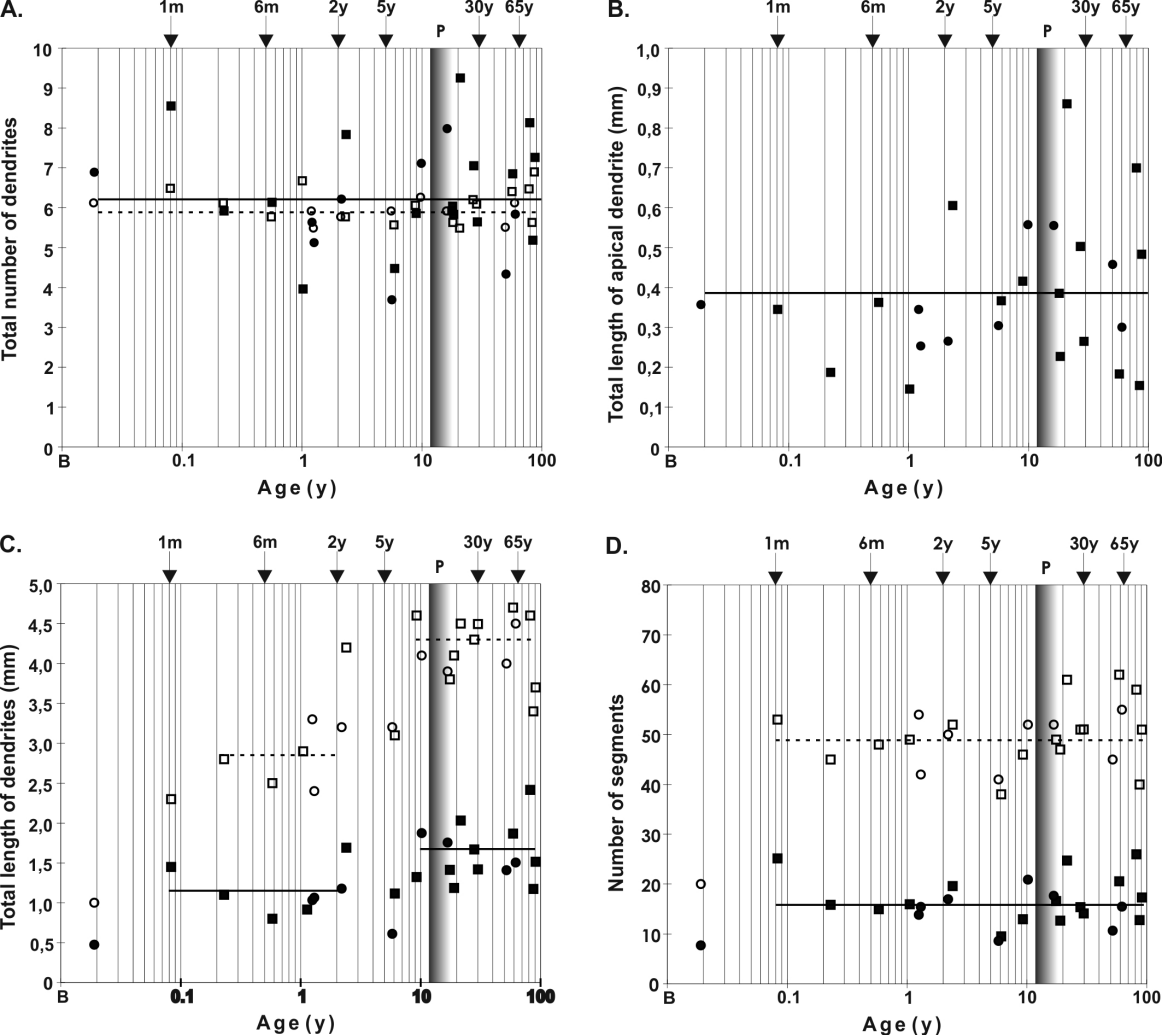
Comparison of basal (open symbol; data from Petanjek et al, 2008 [1]) and apical oblique dendrites (filled symbol) across life-span: (**A**) total number of dendrites, (**B**) total length of apical dendrite main shaft including terminal ramifications, (**C**) total length of oblique and basal dendrites, (**D**) number of segments indicating branching frequency. The temporal growth pattern during the postnatal period (**C**,**D**) does not show major differences between oblique and basal dendrites. Age was presented on a logarithmic scale. Shaded bar represents the period of puberty. Squares represent male subjects; circles represent female subjects. P – puberty; B – birth; m – months; y – years.

**Figure 5:**
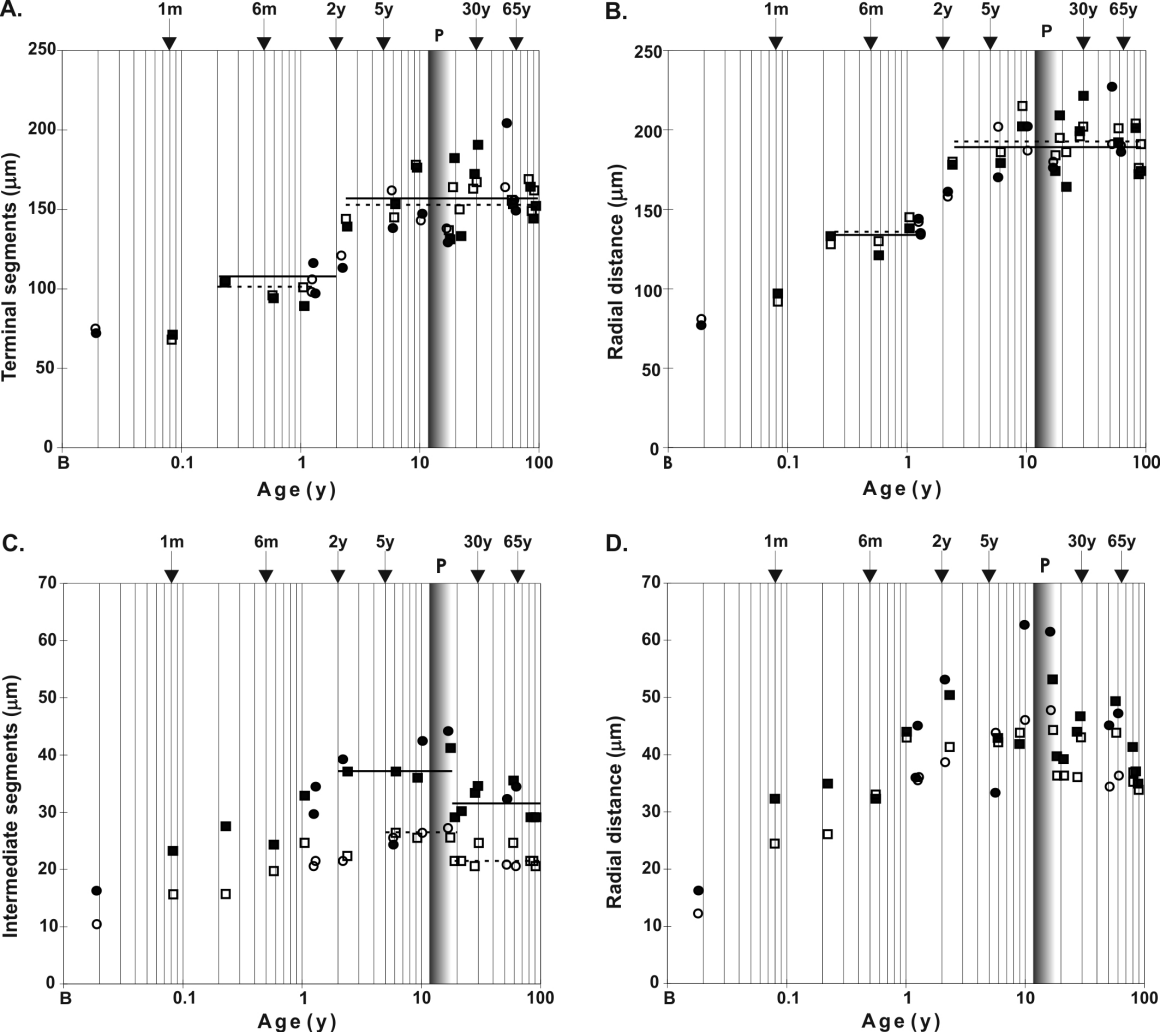
Temporal growth pattern for terminal segments of the layer IIIC pyramidal neurons in Brodmann area 9 during the postnatal period did not differ between oblique (filled symbol) and basal dendrites (open symbol; data from Petanjek et al, 2008 [1]), and analyzed average values in all the age groups were almost the same for terminal segment mean length (**A**) and radial distance (**B**). Transient overgrowth was found on mean length (**C**) and radial distance (**D**) of oblique dendritic intermediate segments. Age was presented on a logarithmic scale. Shaded bar represents the period of puberty. Squares represent male subjects; circles represent female subjects. P – puberty; B – birth; m – months; y – years.

Descriptive analysis revealed large inter-individual differences in the total number of oblique dendrites (Figure 4A) and especially in the average length of apical dendritic main shaft per neuron (Figure 4B). The difference was around 6-fold between the subjects with highest and lowest values (860 µm vs 130 µm; average for all specimens was 380 µm). The total length of oblique dendrites (Figure 4C) and number of segments per neuron (Figure 4D) varied less across subjects: there was a 2- to 2.5-fold difference between the subjects with highest and lowest values. This likely represents a consequence of apical dendrites having been cut at the edge of the section at different parts of their main shaft. In most cases, apical dendrites could not be followed to the beginning of their terminal ramification. On the other hand, the average values of individual intermediate and terminal segments (Figure 5) (not affected by the cutting effect) did not show large inter-individual differences, although the proportion of cut oblique segments was higher than the proportion of cut basal dendritic segments (Table 1).

Statistical analysis showed significant age effect on the total length of oblique dendrites (Figure 4C) and on all variables linked to the individual segment (Figure 5) (see below). The pattern of dendritic growth revealed two growth spurts. The first growth spurt occurred from birth to 1-2.5 months; the second growth spurt occurred between 16-24 months and 2.5 years, and the “dormant” period lasted between 1-2.5 and 16-24 months.

During the first month of life, the total length (Figure 4C) and number of segments of oblique dendrites (Figure 4D) increased around 3 times (from 0.5 to 1.4 mm and from 8.3 to 24.9 segments; *P* < 0.001, t- test). The significant increase in the total length of dendrites (*P* =0.04, ANOVA, post hoc SNK test) detected after the first postnatal month appears to be a direct result of dendritic segments’ elongation, without further branching. Total dendritic length (Figure 4C) did not increase further between 1 and 2.5 months. However, there was an increase in terminal segment length (Figure 5A) and radial distance (Figure 5B), suggesting that further elongation might have occurred between 1 and 2.5 months. Given the length observed at birth, it can be concluded that not more than 25% of the total adult length is achieved prenatally and that roughly 1/3 of elongation takes place in the early postnatal period. The rest of oblique dendritic growth, approximately 1/3 of dendritic length, occurs during the second growth spurt.

In the period from 2.5 months to 2 years, there were no significant differences in the total dendritic length (range 800 to 1200 μm, ANOVA, *post hoc* SNK test) and mean length of individual terminal segments (range 92 to 116 μm) of the oblique dendritic tree. However, considerable increase was detected between 2 and 2.5 years. This second growth spurt was especially evident in terminal segment length values (Figure 5A): all subjects up to 2 years had significantly lower values than all subjects older than 2.5 years (*P* < 0.001, ANOVA, *post hoc* SNK test). The 2.5 months-2 years group also had significantly lower values of both mean length of terminal segments (105 vs 159 μm; *P* < 0.001, *t* test) and total length (1020 vs 1520 μm; *P* < 0.008, *t* test), as well as radial distance of terminal segments (133 μm, range 120-143 μm vs 189 μm, range 163-226 μm, *P* < 0.001, *t* test) (Figure 5B). From 2.5 years onwards, the total length of the oblique dendritic tree fluctuated insignificantly (ANOVA, *post hoc* SNK test) around a stable value. Thus, at 2.5 years oblique dendrites attained their adult-like values, implying that dendrites did not undergo major growth or regressive changes thereafter.

The elongation of individual terminal segments (Figure 5A) and intermediate segments (Figure 5C) displayed different developmental trajectories. The length of individual intermediate segments did not follow the proposed pattern with two growth spurts (Figures 4C, 5A) and increased continuously until the age of 2 years (Figure 5C), when these segments became around 2.5 times longer than at birth. From 19 years onwards, the mean length of individual intermediate segments decreased significantly (*P* = 0.03, ANOVA, *post hoc* SNK test) (Figure 5C). Subjects older than 18 years had significantly lower values of mean dendritic length than subjects 2–17 years old (31.5 μm vs 39.5; *P* < 0.001, *t* test; 5-years-old subject was considered as an outlier and excluded from the analysis). The radial distance showed much higher inter-individual differences (Figure 5D), and statistical analysis confirmed around 20% reduction in the length of intermediate segments (52 vs 42 μm; *P* < 0.006, *t* test; 5-years-old subject was considered as outlier and excluded from the analysis). Taken together, these findings suggest that intermediate segments of L3N cells undergo a period of transient overgrowth between 2 and 17 years of age. Described changes on oblique intermediate segments were not reflected in the values of total dendritic length because their summated length represented just 10-15% of the total length.

### Comparison between oblique and basal dendrites

The developmental trajectories of both basal and oblique dendrites displayed a similar overall pattern but differed in topological and metric variables. Compared to basal dendrites, the oblique dendritic tree consisted of more primary dendrites, which were less bifurcated, that is, less complex.

When comparing the total size and complexity of basal and oblique dendrites, it should be noted that methodological factors in this study affected basal and oblique dendrites differently (Figure 4B). This was particularly reflected in the total number of primary dendrites (Figure 4A), since in most cases one part of oblique dendritic tree was missing from reconstructed neurons. The average number of primary dendrites was similar in oblique and basal dendrites (6.2 vs 6). However, when we compared data from 10 specimens with the highest number of dendrites, the average number of primary oblique dendrites per neuron increased for more than 20%, whereas the average number of primary basal dendrites increased only slightly (3%). This indicates that there were significantly more primary oblique than primary basal dendrites (7.6 vs 6.2; *P* < 0.001, *t* test) per neuron than it could be concluded when we compared the whole sample. In other words, the average number of primary oblique dendrites obtained during the analysis was underestimated, whereas for basal dendrites, it was close to accurate values. Given that the cutting affected the apical main shaft in almost the entire sample, we propose that the accurate average number of primary oblique dendrites is 9 per neuron which is the highest value obtained. If this is correct, neurons generally contain around 50% more primary oblique than primary basal dendrites.

Oblique dendrites were less bifurcated, that is, a single primary oblique dendrite had fewer bifurcation points (and consequently was composed of fewer segments) than a basal dendrite. When we estimated the average number of segments per dendrite and the total number of dendrites, the entire oblique dendritic tree (all side branches included) had on average around 27, and the entire basal dendritic tree had on average around 56 dendritic segments per neuron. In adult specimens the average total length per primary dendrite (including all branches emerging from it) was 2.3 times higher for basal than for oblique dendrites; the estimated average length of an individual oblique dendrite was 350 µm, and that of an individual basal dendrite was 800 μm. Based on these estimates, the total length of oblique dendrites represented around 55%-65% of basal dendrite length, that is, slightly more than 1/3 of the total dendritic tree length out of the apical dendrite main shaft and its terminal ramifications.

Individual intermediate segments of oblique dendrites were, on average, 30% longer than those of basal dendrites during the period of overgrowth both in children and adolescents (39.5 µm vs 26.7 µm; *P* <0.001, *t* test) and adult specimens (31.4 µm vs 22.1 µm, *P* < 0.001, *t* test). In oblique dendrites, the degree-two segments predominated, which are longer than the segments of higher degrees. In oblique dendrites, the average length of all intermediate segments was higher than the average length of degree-two segments on basal dendrites. Therefore, longer intermediate segments on oblique dendrites could not be explained by the predomination of degree-two segments. The length of individual terminal segment was similar in oblique and basal dendrites, both during growth and after the adult values had been achieved (156 µm vs 159 µm), which happens around age of 2.5 years (Figure 5A).

## Discussion

In the present study, we reported two major findings on the postnatal development of L3N in the human DLPFC: (A) the perinatal period represents a phase of rapid dendritic growth for oblique dendrites on L3N in the DLPFC, and (B) L3N display a unique pattern of postnatal oblique dendritic development characterized by two periods of growth separated by a year-long “dormant” stage. This study is a continuation of our previous quantitative analyses (1,10,12-14,32,38,43-45) of prenatal and postnatal dendritic development and life-span changes in the DLPFC from 13.5 postconceptional weeks to 91 years of age.

Previous studies have shown that L3N arrive to the cortical plate at 15 weeks of gestation (4,46,47). For the next 15 weeks, their growth includes the protrusion of primary dendrites (13,44). Their intensive growth begins at around 32 weeks of gestation and coincides with the ingrowth of cortico-cortical glutamatergic afferents into the cortical plate (48-50). It has been suggested that these fibers induce dendritic growth through N-methyl-D-aspartate receptor stimulation (51). Also, the switch of GABA activity from excitatory to inhibitory (52-54) may be responsible for the induction of rapid dendritic growth (55,56).

Perinatal growth spurt of oblique and basal dendrites displays similar overall pattern, characterized first by branching (ie, formation-outgrowth of new dendritic segments) during the first postnatal month, and then by extensive elongation. By the third postnatal month, oblique dendrites had attained 60% of their total adult size and already displayed an adult-like pattern of branching. Although the overall developmental pattern of oblique and basal dendrites is similar, the timing of growth during perinatal period is slightly different. This is not surprising given that basal and apical dendrites respond differently to neurotrophic factors or other molecules, and to neural activity (7,25-28,57,58). The perinatal period is characterized by intensive synaptogenesis. Synaptic density in human and macaque DLPFC reaches the adult levels by the time of birth, but most of the synapses are still immature and dendritic spines are sparse (59). Immature forms of dendritic spines in the human prefrontal cortex mainly disappear by the third postnatal month, and oblique dendrites of the L3N in the DLPFC reach adult spine density around 6 months. These results suggest the presence of an early functioning neural network, established by associative L3N, which may represent a neurobiological basis for developing cognitive functions during the early postnatal stage (60,61).

Both oblique and basal dendrites display a “dormant” period (ie, a temporary growth cessation) after about 3 months, whereas oblique dendrites enter the “dormant” stage a month earlier and remain in it several months longer than basal dendrites. The long “dormant” period and large increase in length (50% of the values at 16 months) occurring so late in the development (around second postnatal year) was not described in previous studies of dendritic development in humans or in other primates at equivalent ages (11-15,62-64). In the typical pattern of dendritic growth (55,56), most of the dendritic field is formed during a short stage of very intensive dendritic growth followed by a much longer period of moderate growth (when around 20% of dendritic size is formed). This final stage of slow dendritic differentiation highly depends on environmental stimulation (65,66) and occurs during a period of most intensive synaptogenesis (55,59). This stage is missing in the L3N, and despite the fact that synaptic spine density continues to increase through the first and second year (59), their growth exhibits a “dormant” period.

One possible explanation of why afferent activity does not induce dendritic growth during this period is the molecular immaturity of L3N, since the intensive molecular maturation of L3N occurred between the ages of 2 and 4 years and is marked by appearance of strong acetylcholinesterase (67) and SMI-32 neurofilament (68) reactivity. Molecular maturation continues through the rest of early childhood, and characteristic intense Nissl staining appears by the age of 5-6 years simultaneously with a temporal overgrowth in the soma size (1). Thus, the second dendritic growth spurt around the age of 2-3 years described here, along with molecular maturation of associative L3N up to the age of 6 years, appears to be related to maturation of their projections. This suggests that changes in the organization of microcircuitries established by L3N represent the biological foundation for the emergence of higher cognitive capacities during the early childhood (69). We propose that associative L3N of the human DLPFC have a central role in producing more efficient network integration across the cerebral cortex. Consequently, developmental changes and maturation of related microcircuitries increase the overall speed and effectivity of cortical network processing (6,16,70,71).

It can be suggested that, due to the central integrative role that L3N play in the cortical circuitries, even small changes in their “quality and quantity” may have important functional implications, with effects on cognitive functioning. It has been documented that a lower socioeconomic status during infancy and early childhood can lead to structural and functional differences in cortical maturation compared to participants of the same age growing up in higher socioeconomic status families (72). Conversely, it has also been suggested that emotional and psychological interaction with the mother enhances theory of mind development in children (73), suggesting that increased social and emotional stimulation may play a role in differentiation and maturation of cortical circuitry. Our assumption is that associative L3N in the DLPFC may be particularly prone to environmental influences, especially during the period of intensive dendritic growth and synaptogenesis in the postnatal stages (74). Selective alterations found on associative neurons in schizophrenia (75), Alzheimer disease (76), neurodevelopmental disorders (29), epilepsy (77), and certain procedures such as callosotomy (78), support this hypothesis.

In addition to the modification of dendritic length and branching, the number of synapses represents another major mechanism for diversification of neuronal connections. It has been proposed that the overproduction rate of synaptic spines is proportional to the level of plasticity (79-81). The largest overproduction has been recorded in the DLPFC of human and nonhuman primates (49). Based on our findings, there is a strong connection between plasticity and synaptic overproduction in the period of early childhood (2.5-6 years), characterized by the highest number of synapses on both oblique and basal dendrites (59). The period of overproduction and elimination on these segments extends into the third decade of life. Such an extraordinarily long phase of developmental reorganization of cortical neuronal circuitry, critical for processing highest mental functions, can help us understand the relationship between the environment and the development of human cognitive and emotional capacities (82,83), as well as the emergence of neuropsychiatric disorders (84).

Our sample consisted of material with a very low PMI, resulting in a sufficient number of Golgi-impregnated neurons to be included in the analysis, surpassing the methodological constrains that often limit the conclusions that can be drawn from the tissue processed using Golgi method. However, the scope of the present study was limited on one subpopulation of neurons – large pyramidal cells in the lower layer III – and it remains to be seen how the age-related changes observed in L3N relate to changes in other classes of neurons in the DLPFC. Given the importance of the prefrontal cortex in human cognition and its structural changes associated with age, further studies should examine whether the observed pattern of changes in dendritic morphology represents a peculiarity of the DLPFC, or it is a feature shared by other prefrontal regions. Functional correlates of these morphological changes should also be explored.

In conclusion, the unique pattern of dendritic growth and spine maturation on both basal and oblique dendrites and the extraordinarily protracted circuitry reorganization up to the post-adolescent period suggests that L3N of the DLPFC are among major elements representing the neurobiological substrate for the appearance of complex cognitive functions and proper socio-emotional maturation. Given the central role of associative L3N in microcircuitries of the DLPFC, alterations of typical dendritic morphology might have important roles in psychiatric disorders characterized by compromised cortico-cortical connectivity, such as autism spectrum disorder (85) and schizophrenia (86). Findings on early intensive differentiation of associative neurons during the perinatal developmental period and the second developmental spurt with protracted circuitry reorganization can be used as the basis for further studies examining, for example, why a pathological event at the pre/perinatal period can manifest years or decades later (84).
